# Arginine vasopressin modulates electrical activity and calcium homeostasis in pulmonary vein cardiomyocytes

**DOI:** 10.1186/s12929-019-0564-3

**Published:** 2019-09-17

**Authors:** Jen-Hung Huang, Yao-Chang Chen, Yen-Yu Lu, Yung-Kuo Lin, Shih-Ann Chen, Yi-Jen Chen

**Affiliations:** 1 Division of Cardiovascular Medicine, Department of Internal Medicine, Wan Fang Hospital, Taipei Medical University, 111 Hsin-Lung Road, Sec. 3, Taipei, 116 Taiwan; 2 Department of Internal Medicine, School of Medicine, College of Medicine, Taipei Medical University, Taipei, Taiwan; 3 Department of Biomedical Engineering, and Institute of Physiology, National Defense Medical Center, Taipei, Taiwan; 4 Division of Cardiology, Department of Internal Medicine, Sijhih Cathay General Hospital, New Taipei City, Taiwan; 5 School of Medicine, Fu-Jen Catholic University, New Taipei City, Taiwan; 6 Heart Rhythm Center and Division of Cardiology, Department of Medicine, Taipei Veterans General Hospital, Taipei, Taiwan; 7 Graduate Institute of Clinical Medicine, College of Medicine, Taipei Medical University, Taipei, Taiwan; 8 Cardiovascular Research Center, Wan Fang Hospital, Taipei Medical University, Taipei, Taiwan

**Keywords:** Atrial fibrillation, Arginine vasopressin, Calcium homeostasis, Pulmonary vein

## Abstract

**Background:**

Atrial fibrillation (AF) frequently coexists with congestive heart failure (HF) and arginine vasopressin (AVP) V1 receptor antagonists are used to treat hyponatremia in HF. However, the role of AVP in HF-induced AF still remains unclear. Pulmonary veins (PVs) are central in the genesis of AF. The purpose of this study was to determine if AVP is directly involved in the regulation of PV electrophysiological properties and calcium (Ca^2+^) homeostasis as well as the identification of the underlying mechanisms.

**Methods:**

Patch clamp, confocal microscopy with Fluo-3 fluorescence, and Western blot analyses were used to evaluate the electrophysiological characteristics, Ca^2+^ homeostasis, and Ca^2+^ regulatory proteins in isolated rabbit single PV cardiomyocytes incubated with and without AVP (1 μM), OPC 21268 (0.1 μM, AVP V1 antagonist), or OPC 41061 (10 nM, AVP V2 antagonist) for 4–6 h.

**Results:**

AVP (0.1 and 1 μM)-treated PV cardiomyocytes had a faster beating rate (108 to 152%) than the control cells. AVP (1 μM) treated PV cardiomyocytes had higher late sodium (Na^+^) and Na^+^/Ca^2+^ exchanger (NCX) currents than control PV cardiomyocytes. AVP (1 μM) treated PV cardiomyocytes had smaller Ca^2+^_i_ transients, and sarcoplasmic reticulum (SR) Ca^2+^ content as well as higher Ca^2+^ leak. However, combined AVP (1 μM) and OPC 21268 (0.1 μM) treated PV cardiomyocytes had a slower PV beating rate, larger Ca^2+^_i_ transients and SR Ca^2+^ content, smaller late Na^+^ and NCX currents than AVP (1 μM)-treated PV cardiomyocytes. Western blot experiments showed that AVP (1 μM) treated PV cardiomyocytes had higher expression of NCX and p-CaMKII, and a higher ratio of p-CaMKII/CaMKII.

**Conclusions:**

AVP increases PV arrhythmogenesis with dysregulated Ca^2+^ homeostasis through vasopressin V1 signaling.

## Introduction

Arginine vasopressin (AVP), is a nonapeptide produced by the posterior pituitary gland, mainly synthesized and secreted in the hypothalamus. AVP binds to its receptor to promote vasoconstriction through modulation of adenosine triphosphate-sensitive potassium channel function and nitric oxide (NO) production, and enhances the vascular response to catecholamines [1]. AVP is an essential stress hormone released in response to hyperosmolarity, hypotension, or hypovolemia [2]. AVP has several physiological functions, including V1a receptor-mediated regulation of blood pressure and V2 receptor-mediated control of body water [3]. AVP has been clinically proven to be related to the severity of heart failure (HF) [4, 5]. AVP V2 receptor antagonists are also used to treat hyponatremia associated with HF [6]. OPC 41061, AVP V2 receptor antagonist, had been demonstrated to suppress multiple potassium currents on GH3 (a rat clonal pituitary cell line) [7]. Additionally, AVP is a nonsympathomimetic vasopressor, which causes vasoconstriction via activation of the V1 vasopressin receptor [8]. The V1a receptor, associated with a G_q/11_ protein, hydrolyses phosphatidylinositol 4,5-bisphosphate into inositol trisphosphate (IP_3_) and diacylglycerol (DAG) via the activation of phospholipase C [9]. IP_3_ induces calcium (Ca^2+^) release from the sarcoplasmic reticulum (SR) [10, 11], whereas DAG activates protein kinase C, which opens voltage-gated Ca^2+^ channels and closes potassium channels [11].

HF and atrial fibrillation (AF) are closely interrelated with sharing similar risk factors and pathophysiology [12]. Patients with concomitant HF and AF suffer from even worse symptoms and poorer prognosis. Centrally liberated AVP increases sympathetic outflow to the cardiovascular system and may increase the risk of arrhythmia and sudden death [13]. However, the relationship between AF and AVP has not been clearly elucidated. Earlier studies showed that increased AF occurrence in patients with postoperative vasoplegic shock more frequently experienced new AF with prolonged AVP therapy [14, 15].

Pulmonary vein (PV) myocardium consists of a mixture of working cardiomyocytes and pacemaker cells. It plays a critical role in the genesis and maintenance of AF [16]. PVs are important sources of ectopic beats that can initiate paroxysmal AF and ectopic atrial tachycardia [17]. PV cardiomyocytes exhibit distinct electrophysiological characteristics that include spontaneous activity and triggers, which may contribute to PV arrhythmogenesis [18]. Abnormal Ca^2+^ regulation plays a pivotal role in PV electrical activity. Late sodium currents (I_Na-late_) and intracellular Ca^2+^ (Ca^2+^_i_) dynamics play an important role in PV arrhythmogenesis and HF. Dysregulated Ca^2+^ homeostasis, and increased Ca^2+^ spark frequency promotes arrhythmogenesis of PV cardiomyocytes in HF, which may play an important role in the development of AF [19]. Interestingly, in vitro and in vivo studies showed that AVP produces relatively less vasoconstriction in pulmonary circulation [20, 21]. However, the role of AVP in PV arrhythmogenesis was not clear. Since AVP signaling may modulate Ca^2+^ homeostasis, we suggest that enhanced AVP might contribute to PV arrhythmogenesis. The aim of this study was an evaluation of the effects of AVP on the PV electrophysiological characteristics and Ca^2+^ handling and an investigation of the potential mechanisms.

## Materials and methods

### Isolation of single cardiomyocytes and cell preparations

The investigation was approved by the local ethics review board and conformed to the institutional Guide for the Care and Use of Laboratory Animals published by the US National Institutes of Health. Male rabbits (2.0~3.0 kg) were euthanized using intramuscular injections of a mixture of Zoletil 50 (10 mg/kg) and xylazine (5 mg/kg) with an overdose of isoflurane (5% in oxygen) from a precision vaporizer. Single cardiomyocytes from rabbit PVs were enzymatically dissociated through a previously described procedure [18]. In brief, a mid-line thoracotomy was performed, and the heart and lungs were removed. PVs were perfused in a retrograde manner via polyethylene tubing cannulated through the aorta and left ventricle into the left atrium. The free end of the polyethylene tube was connected to a Langendroff perfusion column for perfusion with oxygenated normal Tyrode’s solution (containing (in mM): NaCl 137, KCl 5.4, CaCl_2_ 1.8, MgCl_2_ 0.5, HEPES 10 and glucose 11; with pH adjusted to 7.4 by titration with 1 N NaOH. The perfusate was replaced with oxygenated Ca^2+^-free Tyrode’s solution containing 300 units/ml collagenase (Sigma Type I) and 0.25 units/ml of protease (Sigma, Type XIV) for 8~12 min. Proximal PVs were cut away from the atrium and lung, and were gently shaken in 5~10 ml of Ca^2+^-free oxygenated Tyrode’s solution until single cardiomyocytes were obtained. The solution was then gradually changed to oxygenated normal Tyrode’s solution. Cells were allowed to stabilize in the bath for at least 30 min before the experiments were started. Single cardiomyocytes with spontaneous activity were identified by the presence of constant beating during perfusion with Tyrode’s solution. PV cardiomyocytes were incubated without (control) and with AVP (0.1 and 1 μM), AVP (1 μM) combined with or without OPC 21268 (AVP V1 receptor antagonist, 0.1 μM), OPC 41061 (AVP V2 receptor antagonist, 10 nM) [22, 23] for 4~6 h before patch clamp or western blot.

### Patch clamp experiments

A whole-cell patch-clamp was used to record ionic currents and action potentials (APs) in isolated PV cardiomyocytes with an Axopatch 1D amplifier (Axon Instruments, Foster City, CA, USA) at 35 °C ± 1 after rupture or perforation (for L-type Ca^2+^ current, I_Ca-L_) at an approximately similar period, as previously described [18]. The micropipette resistance was 3~5 MΩ. A small hyperpolarizing step from a holding potential of − 50 mV to a testing potential of − 55 mV for 80 ms was delivered at the start of each experiment. The area under the capacitive current curve was divided by the applied voltage step to obtain the total cell capacitance. Normally, 60%~ 80% series resistance (Rs) was electronically compensated. APs were recorded in the current-clamp mode and ionic currents in the voltage-clamp mode. Spontaneous beating rate was defined as the constant occurrence of spontaneous APs in the absence of any electrical stimuli. APs were analyzed for maximum diastolic potential (MDP), amplitude (APA), threshold potential (the inflection point on the rising phase of action potential) [24, 25], early and late diastolic depolarization (linear and non-linear components of the interval between MDP and threshold potential) [26, 27], and AP duration (APD) at 75, 50 and 20% repolarization of the amplitude (APD_75_, APD_50_, and APD_20_) [28].

Micropipettes were filled with a solution containing (in mM) CsCl 130, MgCl_2_ 1, MgATP 5, HEPES 10, Na_2_GTP 0.1, and Na_2_ phosphocreatine 5, titrated to a pH of 7.2 with CsOH for the experiments on the I_Ca-L_; with a solution containing (in mM) NaCl 20, CsCl 110, MgCl_2_ 0.4, CaCl_2_ 1.75, tetraethylammonium chloride (TEA-Cl) 20, BAPTA 5, glucose 5, MgATP 5, and HEPES 10, titrated to a pH of 7.25 with CsOH for experiments on the Na^+^/ Ca^2+^ exchanger (NCX) current; and with a solution containing (in mM) CsCl 130, Na_2_ATP 4, MgCl_2_ 1, EGTA 10, and HEPES 5 at a pH of 7.3 with NaOH for the I_Na-Late_; with a solution containing (in mM) KCl 20, K aspartate 110, MgCl_2_ 1, MgATP 5, HEPES 10, EGTA 0.5, Na_2_GTP 0.1, and Na_2_ phosphocreatine 5, titrated to a pH of 7.2 with KOH for experiments on the AP.

The I_Na-Late_ was recorded at room temperature with an external solution containing (in mM): NaCl 130, CsCl 5, MgCl_2_ 1, CaCl_2_ 1, HEPES 10, and glucose 10 at a pH of 7.4 with NaOH by a step/ramp protocol (− 100 mV stepped to + 20 mV for 100 ms, then ramped back to − 100 mV over 100 ms) to enhance I_Na-Late_ since the non-equilibrium gating gives rise to a fast recovery of the Na^+^ channel from inactivated state and reactivation during repolarization ramp [29]. The external solution containing CaCl_2_ has been shown to block all movement of monovalent ions through Na^+^ channels, which may avoid persistent Ca^2+^ current through Na^+^ channels [30]. The application of tetrodotoxin (30 μM) results inhibition of larger fraction of the plateau current of the Na^+^ current, which has been shown to dissect the full measure of I_Na-Late_ [31]. The I_Na-late_ was measured as the tetrodotoxin (30 μM)-sensitive part of the current traces obtained when the voltage was ramped back to − 100 Mv [32].

The I_Ca-L_ was measured as an inward current during depolarization from a holding potential of − 50 mV to test potentials ranging from − 40 to + 60 mV in 10 mV steps for 300 ms at a frequency of 0.1 Hz using a perforated patch clamp with amphotericin B. NaCl and KCl in the external solution were replaced with TEA-Cl and CsCl. To avoid run-down effect (the spontaneous decrease of voltage-activated current after onset of dialysis by the pipette solution) [33], the I_Ca-L_ was measured at 5~15 min after perforating the membrane patch in each PV cardiomyocyte.

The NCX current was elicited by test pulses of between − 100 and + 100 mV from a holding potential of − 40 mV for 300 ms at a frequency of 0.1 Hz. The amplitudes of the NCX current were measured as 10 mM nickel-sensitive currents. The external solution (in mM) consisted of NaCl 140, CaCl_2_ 2, MgCl_2_ 1, HEPES 5, and glucose 10 with a pH of 7.4, and contained strophanthidin (10 μM), nitrendipine (10 μM), and niflumic acid (100 μM).

### Measurement of intracellular and SR calcium content

As described previously, PV cardiomyocytes were loaded with fluorescent Ca^2+^ (10 μM, fluo-3/AM) for 30 min at room temperature [34]. The Fluo-3 fluorescence was excited using the 488-nm line of an argon ion laser and emission was recorded at > 515 nm. Cells were repetitively scanned at 2-ms intervals. Fluorescence imaging was performed with a laser scanning confocal microscope (Zeiss LSM 510, Carl Zeiss, Jena, Germany) and an inverted microscope (Axiovert 100, Carl Zeiss). The fluorescent signals were corrected for variations in dye concentrations by normalizing the fluorescence (F) against the baseline fluorescence (F_0_), to obtain reliable information about transient intracellular Ca^2+^ (Ca^2+^_i_) changes from baseline values (F - F_0_ (ΔF)/F_0_) and to exclude variations in the fluorescence intensity by different volumes of injected dye. The Ca^2+^_i_ transient, peak systolic Ca^2+^_i_, and diastolic Ca^2+^_i_ were measured during spontaneous beating and pacing with a 2-Hz field-stimulation with 10-ms twice-threshold strength square-wave pulses. The SR Ca^2+^ content was estimated by the rapid addition of 20 mM caffeine after electric stimulation at 2 Hz for at least 30 s. The total SR Ca^2+^ content was determined from the peak amplitude of the caffeine-induced Ca^2+^_i_ transients.

For the measurement of SR Ca^2+^ leak, PV cardiomyocytes were incubated with AVP (1 μM) or AVP (1 μM) plus KN-93 (a Ca^2+^/calmodulin-dependent protein kinase II, CaMKII inhibitor, 1 μM) for 4~6 h before experiments. PV cardiomyocytes were stimulated at 2 Hz in normal Tyrode’s solution to bring the cellular Ca^2+^ content to a steady state. In the control condition, Ca^2+^_i_ was monitored while 0 Na^+^, 0 Ca^2+^ Tyrode’s solution (without tetracaine) was perfused for a minimum of 20 s. In the test condition, the superfusate was rapidly switched to 0 Na^+^, 0 Ca^2+^ Tyrode’s solution with 1 mM tetracaine after the last pulse for a minimum of 20 s. Under this condition, the ryanodine receptor (RyR) is abruptly blocked and the Ca^2+^ leak can be measured as a drop in Ca^2+^_i_.

### Western blot analysis

Control and AVP (1 μM)-treated PV cardiomyocytes were centrifuged and washed with cold PBS, and lysed on ice for 30 min in RIPA buffer containing 50 mM Tris, pH 7.4, 150 mM NaCl, 1% NP40, 0.5% Na^+^ deoxycholate, 0.1% Na^+^ dodecyl sulfate (SDS), and protease inhibitor cocktails (Sigma-Aldrich, St Louis, MO, USA). The protein concentration was determined using Bio-Rad protein assay reagent (Bio-Rad, Hercules, CA, USA). Proteins were separated on 4%~ 12% SDS-polyacrylamide gel by electrophoresis (PAGE) under reducing conditions and electrophoretically transferred to an equilibrated polyvinylidene difluoride membrane (Amersham Biosciences, Buckinghamshire, UK). All blots were probed with primary antibodies against NCX (Swant, Bellinzona, Switzerland), Ca^2+^/calmodulin-dependent protein kinase II (CaMKII; Abcam, Cambridge, UK), pCaMKII at Thr 286, glyceraldehyde-3-phosphate dehydrogenase (GAPDH) (MBL, Nagoya, Japan), AVP V1a receptor (AVPR1a, Biorbyt, Riverside, UK), AVP V2 receptor (AVPR2, Abbexa, Cambridge, UK), and all secondary antibodies conjugated with horseradish peroxidase. All bound antibodies were detected with an enhanced chemiluminescence detection system (Millipore, Billerica, MA, USA) and analyzed with AlphaEaseFC software. All targeted bands were normalized to GAPDH to confirm equal protein loading.

### Data and statistical analysis

All continuous parameters were expressed as mean ± the standard error of the mean (SEM). An unpaired t-test, one-way or two-way analysis of variance (ANOVA) with a Duncan post-hoc test was used to compare differences between the groups. A *P* value of < 0.05 was considered statistically significant.

## Results

### Effects of AVP and AVP receptor antagonists on PV electrical activity, and AVP receptor expressions on PV cardiomyocytes

**Fig. 1:**
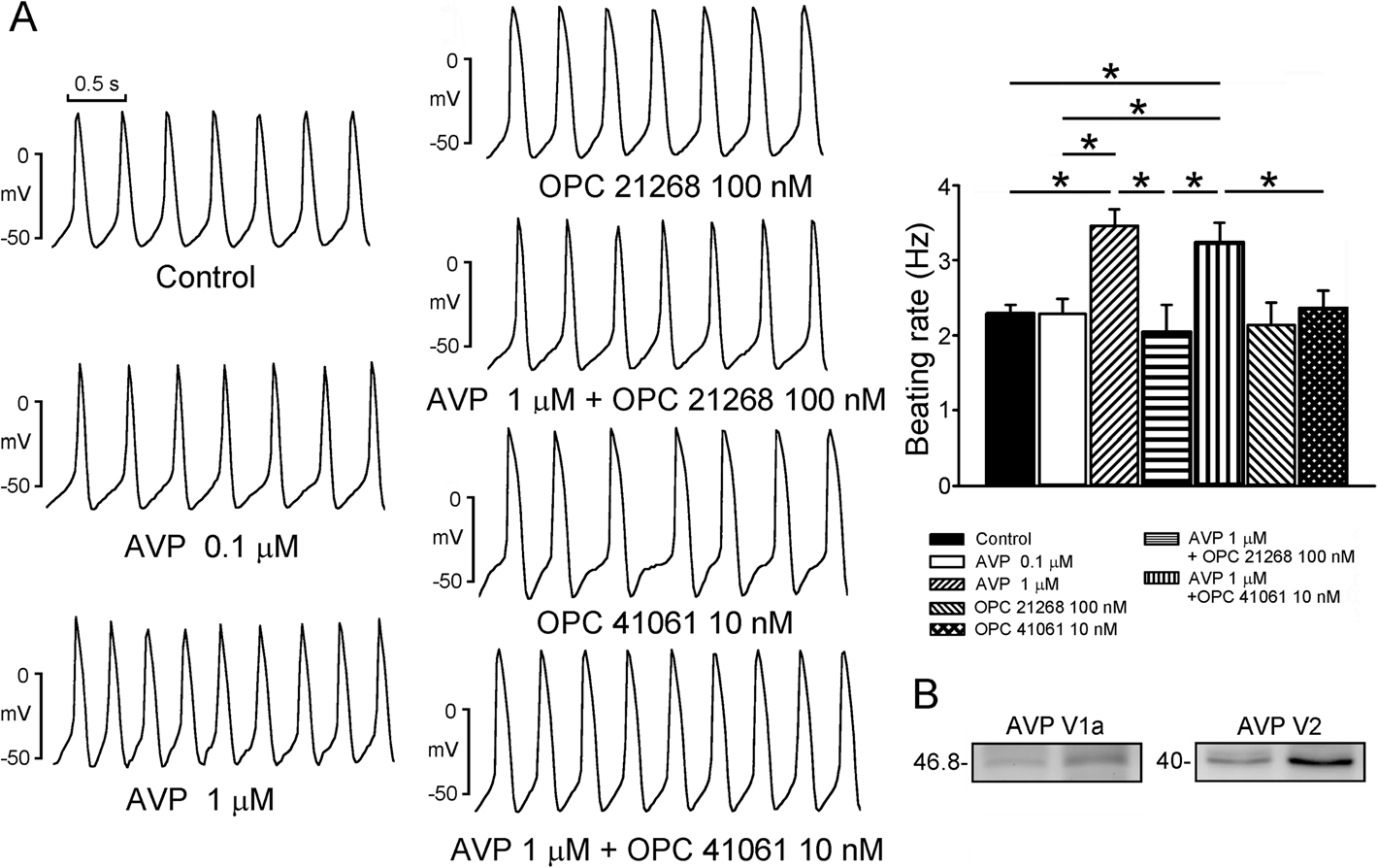
Effects of arginine vasopressin (AVP) and its antagonist OPC 21268 and OPC 41061 on the spontaneous activity of pulmonary vein (PV) cardiomyocytes. **a** Examples and average data of spontaneous activity from control (*n* = 12) and from PV cardiomyocytes treated with either AVP (0.1 μM, *n* = 11), AVP (1 μM, *n* = 13), OPC 21268 (0.1 μM, *n* = 10), AVP (1 μM) plus OPC 21268 (0.1 μM) (*n* = 11), OPC 41061 (10 nM, *n* = 10), or AVP (1 μM) plus OPC 41061 (10 nM) (*n* = 12). **b** Expression of AVP V1a receptor (AVPR1a) and V2 receptor (AVPR2) on PV cardiomyocytes. *P < 0.05

As shown in Fig. 1a, AVP (0.1 and 1 μM)-treated PV cardiomyocytes had a faster dose dependent beating rate than control PV cardiomyocytes by 4 and 37% respectively. AVP (1 μM)-treated PV cardiomyocytes had a greater slope of late diastolic depolarization and a shorter beating rate interval than other groups. The AP features, threshold potential, and the slope of early diastolic depolarization of PV cardiomyocytes were similar among different groups (Table 1).

**Table 1:** Action potential (AP) parameters of pulmonary vein cardiomyocytes treated with or without (control) arginine vasopressin (AVP) and/or AVP receptor antagonist (V1,OPC 21268 and V2 OPC 41061)

	APA (mV)	APD_75_ (msec)	APD_50_ (msec)	APD_20_ (msec)	Beating rate interval (msec)	EDD (mV/msec)	LDD (mV/msec)	MDP (mV)	TP (mV)
Control, (*n* = 12)	80 ± 6	140 ± 13	99 ± 13	54 ± 8	453 ± 28	0.13 ± 0.05	0.21 ± 0.04	−55 ± 4	−39 ± 3
AVP (0.1 μM), (*n* = 11)	82 ± 8	164 ± 18	109 ± 16	65 ± 11	432 ± 35^#^	0.08 ± 0.01	0.33 ± 0.05^#^	−60 ± 2	−32 ± 5
AVP (1 μM), (*n* = 13)	71 ± 7	121 ± 12	82 ± 8	43 ± 5	305 ± 21*	0.09 ± 0.01	0.43 ± 0.06*	−53 ± 3	−32 ± 2
AVP (1 μM) + OPC 21268 (0.1 μM), (*n* = 11)	80 ± 6	131 ± 8	91 ± 7	48 ± 4	813 ± 213^#^	0.08 ± 0.01	0.25 ± 0.03^#^	−56 ± 4	−36 ± 3
AVP (1 μM) + OPC 41061 (10 nM), (*n* = 12)	90 ± 4	142 ± 4	104 ± 3	58 ± 4	329 ± 22*	0.11 ± 0.02	0.37 ± 0.02*	−54 ± 5	−36 ± 3
OPC 21268 (0.1 μM), (*n* = 10)	90 ± 4	148 ± 13	97 ± 10	57 ± 8	581 ± 100	0.09 ± 0.02	0.29 ± 0.03	−62 ± 3	−34 ± 3
OPC 41061 (10 nM), (*n* = 10)	94 ± 4	158 ± 9	118 ± 8	70 ± 5	468 ± 52^§^	0.09 ± 0.01	0.24 ± 0.04^§^	−59 ± 2	−30 ± 3

APD_75_, APD_50_, and APD_20_ = AP duration at 75, 50 and 20% repolarization of the amplitude, *EDD* Early diastolic depolarization, *LDD* Late diastolic depolarization, *MDP* Maximum diastolic potential, *TP* Threshold potential. ^*^ *P* < 0.05 vs Control, ^#^ *P* < 0.05 vs AVP (1 μM), ^§^ *P* < 0.05 OPC 41461 (10 nM) vs AVP (1 μM) + OPC 41461 (10 nM),

The beating rate in OPC 21268 (0.1 μM) or OPC-41061 (10 nM)-treated PV cardiomyocytes was similar to that in control PV cardiomyocytes. However, combined OPC 21268 (0.1 μM) and AVP (1 μM)-treated PV cardiomyocytes had similar beating rate and the slope of late diastolic depolarization as compared to the control (Table 1), suggesting that OPC 21268 (0.1 μM) may attenuate the effects of AVP on PV electrical activity. The beating rate in combined OPC 41061 (10 nM) and AVP (1 μM)-treated PV cardiomyocytes was similar to that in AVP (1 μM)-treated PV cardiomyocytes. This finding suggests that OPC 41061 (10 nM) did not change the electrophysiological effects of AVP on PV cardiomyocytes (Fig. 1a). Moreover, western blot expressions showed that both AVP V1 and V2 receptors were expressed in rabbit PV cardiomyocytes (Fig. 1b).

### Effect of AVP and AVP receptor antagonists on ionic currents of PV cardiomyocytes

**Fig. 2:**
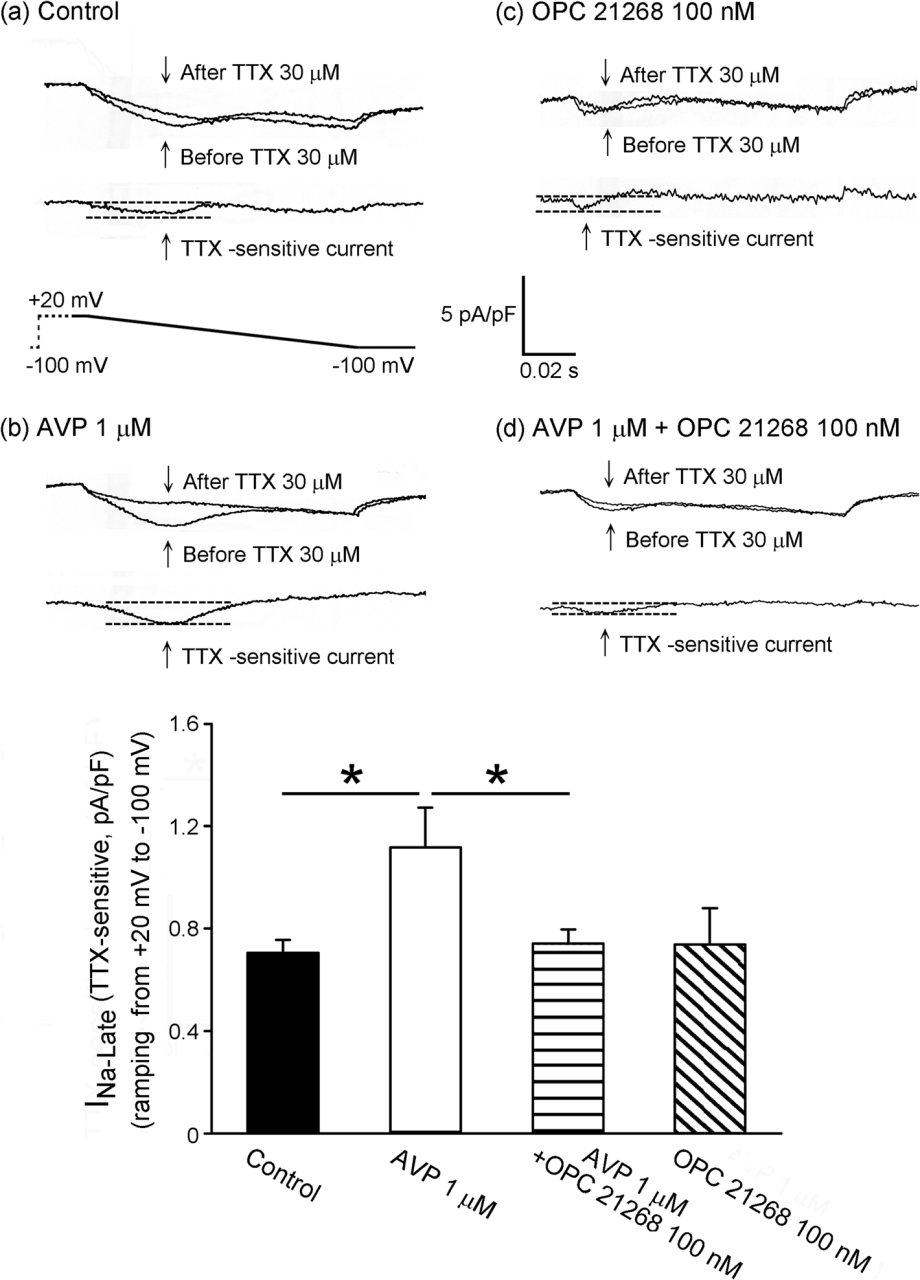
Effects of arginine vasopressin (AVP) on the late sodium current (I_Na-Late_) in pulmonary vein (PV) cardiomyocytes with and without AVP (1 μM) or OPC 21268 (0.1 μM). An example and the average data of the I_Na-Late_ from (**a**) control (*n* = 12) and from PV cardiomyocytes treated with either (**b**) AVP (1 μM, *n* = 12), (**c**) OPC 21268 (0.1 μM, *n* = 9), or (**d**) AVP (1 μM) plus OPC 21268 (0.1 μM) (*n* = 11). I_Na-Late_ was measured as the tetrodotoxin (TTX)-sensitive current during ramp pulse from + 20 mV to − 100 mV. *P < 0.05

**Fig. 3:**
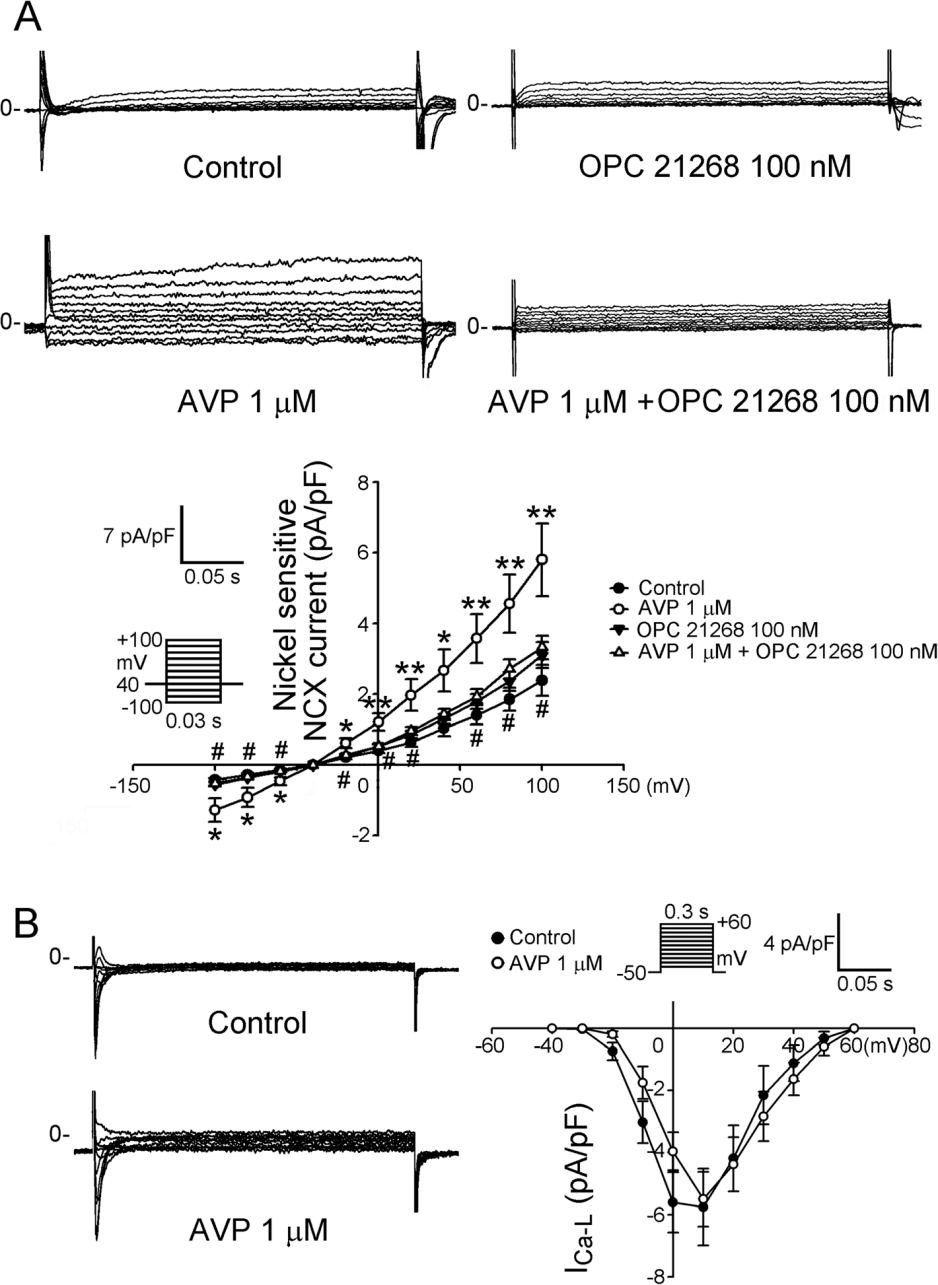
Effects of arginine vasopressin (AVP) on the sodium/calcium exchanger (NCX), and L-type calcium (I_Ca-L_) currents in pulmonary vein (PV) cardiomyocytes with and without AVP (1 μM) or OPC 21268 (0.1 μM). **a** An example and the average data of the NCX from control (*n* = 10) and PV cardiomyocytes treated with either AVP (1 μM, *n* = 10), OPC 21268 (0.1 μM, *n* = 9), or AVP (1 μM) plus OPC 21268 (0.1 μM) (*n* = 10). **b** An example and the average data of the I_Ca-L_ from control (*n* = 9) and AVP (1 μM)-treated (*n* = 12) PV cardiomyocytes. The inset in the current traces shows the clamp protocol. * P < 0.05 and **P < 0.01 Control vs AVP (1 μM); #P < 0.05 AVP (1 μM) vs AVP (1 μM) combined with OPC 21268 (0.1 μM

Figure 2 shows that AVP (1 μM)-treated PV cardiomyocytes had a 58% larger I_Na-Late_ than the control cells. As shown in Fig. 3, AVP (1 μM)-treated PV cardiomyocytes had larger increases in the forward and reverse modes of NCX current (by 202% in the peak forward and 143% in the peak reverse mode current elicited from − 40 to − 100 mV). However, control and AVP (1 μM)-treated PV cardiomyocytes had similar I_Ca-L_. Compared to the control, OPC 21268 (0.1 μM) did not change the current density of I_Na-Late_ and NCX of PV cardiomyocytes. However, OPC 21268 (0.1 μM) can reverse the effects of AVP (1 μM) on I_Late-Na_ and NCX of PV cardiomyocytes.

### Effects of AVP on calcium homeostasis

**Fig. 4:**
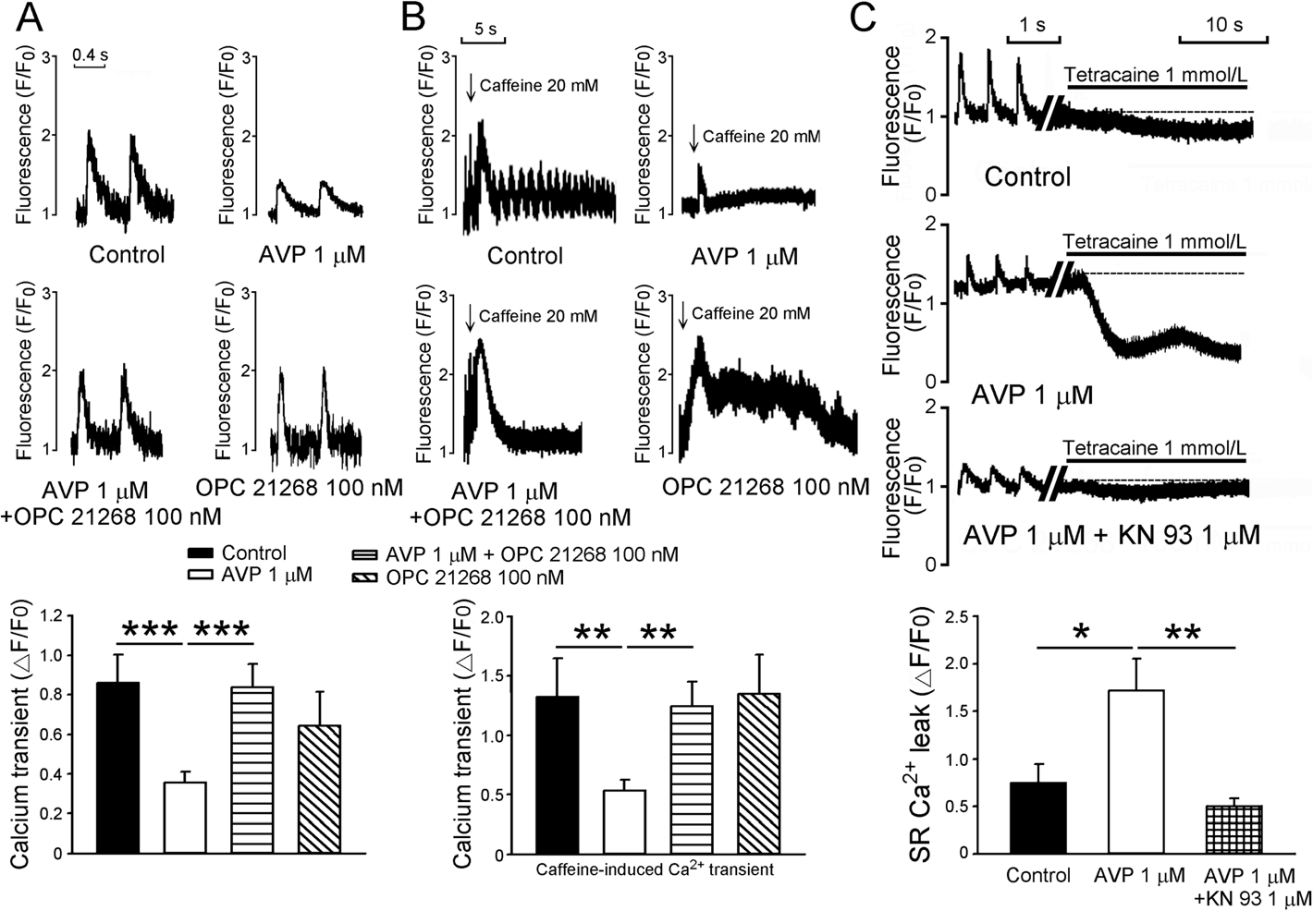
Effects of arginine vasopressin (AVP) and its antagonist OPC 21268 on intracellular calcium (Ca^2+^) homeostasis and sarcoplasmic reticulum (SR) Ca^2+^ leak in pulmonary vein (PV) cardiomyocytes. **a** An example and average data of Ca^2+^_i_ transients from control (*n* = 20), AVP (1 μM)-treated (*n* = 22), AVP (1 μM) combined with OPC 21268 (0.1 μM)-treated (*n* = 25), and OPC 21268 (0.1 μM)-treated (*n* = 11) PV cardiomyocytes. **b** An example and average data of caffeine-induced Ca^2+^_i_ transients in control (*n* = 13) and PV cardiomyocytes treated with either AVP (1 μM, *n* = 15), AVP (1 μM) plus OPC 21268 (0.1 μM) (*n* = 21), or OPC 21268 (0.1 μM, *n* = 11). **c** An example and average data of SR Ca^2+^ leak from control (*n* = 14), AVP (1 μM)-treated (*n* = 16), and AVP (1 μM) combined with KN-93 (1 μM)-treated (*n* = 10) PV cardiomyocytes. *P < 0.05; **P < 0.01; ***P < 0.005

As can be seen in Fig. 4, AVP (1 μM)-treated PV cardiomyocytes had smaller Ca^2+^ transients and caffeine-induced Ca^2+^ transients than the control by 59 and 60%, which suggests they had stored less Ca^2+^. Similarly, spontaneous Ca^2+^ transients in AVP (1 μM)-treated PV cardiomyocytes (*n* = 20) were smaller than those in the control (*n* = 22) by 67% (*P* < 0.001). OPC 21268 (0.1 μM)-treated PV cardiomyocytes combined with or without AVP (1 μM) had similar Ca^2+^ transients and caffeine-induced Ca^2+^ transients to the control, suggesting that the effects of AVP (1 μM) on Ca^2+^ transients and SR Ca^2+^ content can be attenuated by OPC 21258. Moreover, AVP (1 μM)-treated PV cardiomyocytes had greater Ca^2+^ leak than the control, which was attenuated by the presence of KN-93 (1 μM).

**Fig. 5:**
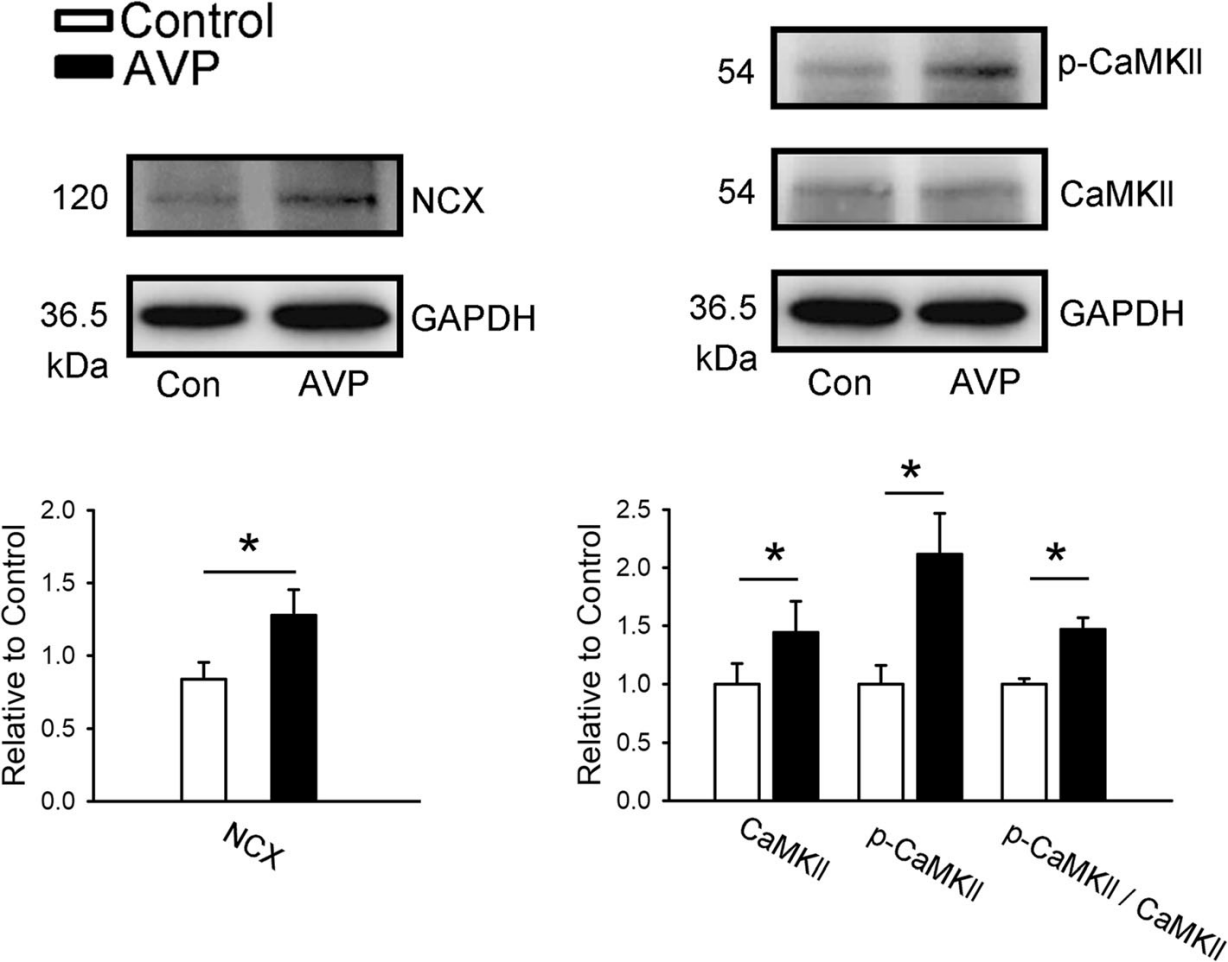
Effects of arginine vasopressin (AVP) on sodium (Na^+^)/calcium (Ca^2+^) exchanger (NCX) and Ca^2+^/calmodulin-dependent protein kinase II (CaMKII) in pulmonary vein (PV) cardiomyocytes. Representative immunoblot and average data of Na^+^/Ca^2+^ exchanger (NCX), Ca^2+^/calmodulin-dependent protein kinase II (CaMKII), and phosphorylated ratio of CaMKII from control (*n* = 6) and AVP (1 μM)-treated PV cardiomyocytes (*n* = 6). *P < 0.05

AVP (1 μM)-treated PV cardiomyocytes had larger protein expressions of NCX and p-CaMKII, but the protein expression of CaMKII was similar between the control and AVP (1 μM)-treated PV cardiomyocytes (Fig. 5). Compared to control, the ratios of p-CaMKII/CaMKII were increased in AVP (1 μM)-treated PV cardiomyocytes.

## Discussion

In this study, it was found for the first time, that AVP has direct effects on electrical activity and Ca^2+^ homeostasis in PV cardiomyocytes. AVP increased the PV beating rate with a dose-dependent response. The increased PV beating rate caused by AVP can be ameliorated by V1 receptor antagonist OPC 21268. OPC 21268 did not change PV electrophysiological properties or Ca^2+^ homeostasis, suggesting that AVP directly regulates PV spontaneous activity via V1 receptor signaling. Previous study had shown that OPC 41061 displayed an inhibitory effect on AVP-induced cAMP increasing and modulated intracellular Ca^2+^ at the concentration of 10 nM [23]. The IC_50_ values for OPC 41061-mediated inhibition of voltage-gated potassium currents ranged between 2 and 7 μM in GH3 cells derived from rat pituitary tumors [7]. In addition, OPC 41061 at higher concentrations may have biological effects in renal ciliary function independent of its binding to V2 receptor [35]. Therefore, the presence of these vasopressin antagonists could potentially exert direct perturbations on the functional activities in different types of cells. The effect of vasopressin antagonists on PV arrhythmogenesis in vivo still remains to be further investigated. Previous studies have shown that AVP V1a and V2 receptor were expressed in human and rodent hearts [36–38]. Similarly, by western blot, this study found both AVP V1a and V2 receptors were expressed in rabbit PV cardiomyocytes. However, V2 receptor antagonist did not change the effects of AVP on PV spontaneous activity, suggesting that V2 receptor signaling may not contribute to the electrical effects of AVP on PV cardiomyocytes. Previous study was found that AVP at 1 μM mobilized 60% of cell-associated Ca^2+^ and decreased protein synthesis by 50% within 20–30 min [39]. Since the concentration (1 μM) used in this study was clinically relevant [39], our findings suggest that AVP may increase the potential risk of AF by escalating PV arrhythmogenesis.

We previously found that enhanced I_Na-Late_ increases PV arrhythmogenesis [17]. In the present study, it was found that AVP-treated PV cardiomyocytes showed larger I_Na-Late_ and NCX than control PV cardiomyocytes. There are different methods of measuring the I_Na-Late_ [40, 41]. To dissect out I_Na-Late_ to a greater degree, we applied high concentration of tetrodotoxin (30 μM) to block Na^+^ current during a repolarizing voltage ramp [29, 31], which was expected to enhance I_Na-Late_. Increasing I_Na-Late_ plays an important role in PV arrhythmogenesis by reducing the repolarization reserve. This opposes the repolarizing potassium currents and delays repolarization [42]. A reduction of the repolarization reserve in PV cardiomyocytes with elevated I_Na-Late_ is more likely to develop early afterdepolarization in response to triggers [42]. I_Na-Late_ would result in an increase of intracellular Na^+^ concentration, which would activate reversed NCX [17], subsequently inducing the genesis of triggered activity [43]. We found that AVP-treated PV cardiomyocytes had increasing NCX, which may be caused by increasing I_Na-Late_ in addition to the increasing effect of AVP on NCX protein.

Cardiac Ca^2+^ homeostasis plays a crucial role in the maintenance of cardiac excitation and contraction, and significantly regulates cardiac contractility. Inhomeostasis of intracellular Ca^2+^_i_ is common in a number of pathological conditions and contributes to arrhythmogenicity [44, 45]. The most recognized action of NCX is its Ca^2+^ removal function in forward mode at membrane voltages less than the equilibrium potential [46]. AVP enhanced forward mode NCX, which caused Ca^2+^ efflux, and resulted in a decrease of Ca^2+^_i_ transients. This decrease in Ca^2+^_i_ transients by AVP may contribute to the known adverse cardiac effects after AVP treatment [47]. Counteraction of the effects of AVP on Ca^2+^_i_ transients and SR Ca^2+^ content in PV cardiomyocytes by the antagonist OPC 21268, suggests that this effect is mainly V1 signal dependent. Previous study has found that AVP can elicit Ca^2+^ entry through a receptor-mediated Ca^2+^-membrane non-selective cation channel in aortic smooth muscle cells, which regulates smooth muscle contractility and enhances vascular tone. OPC 21268 was noted to reverse AVP-induced activation of nonselective cation currents in aortic smooth muscle cells [48]. PVs contain vascular structure and cardiomyocytes. Previous studies have shown that stretch increased PV arrhythmogenesis through mechano-electrical feedback [49]. Therefore, Ca^2+^ influx through vasopressin-induced nonselective cation currents of PV smooth muscle cells may increase vascular stretch, further increasing PV arrhytmogenesis in vivo. In addition, the activity of vascular smooth muscle cells may influence the membrane potential of PV cardiomyocytes via intercellular transfer of electrical signals occurring between PV cardiomyocytes and vascular smooth myocytes of PVs, thereby exacerbating the propensity of PV arrhythmogenesis or cardiac dysrhythmias. Moreover, several previous studies have demonstrated the ability of caffeine to activate intermediate-conductance Ca^2+^-activated potassium channels [50–52], which are also functionally expressed in PV cardiomyocytes [53]. Therefore, part of caffeine-mediated changes in cytosolic Ca^2+^ transient could be secondarily attributed to its activation of these channels.

SR Ca^2+^ leak, the release of small amounts of Ca^2+^, occurs when altered RyR spontaneously opens in diastole [54]. Diastolic Ca^2+^ release activates the forward mode of NCX current on the late diastolic depolarization. This late diastolic depolarization acceleration by NCX is required for the subsequent timely rapid AP upstroke [55, 56]. In the present study, AVP increased forward mode of NCX as well as SR Ca^2+^ leak resulting steepness of late diastolic potential, which could be responsible for increasing automaticity [57]. Blocking AVP V1 receptor attenuated AVP-increased PV spontaneous activity, NCX and late diastolic potential, which implies AVP V1 receptor may play a role in AVP-increased PV spontaneous activity. In HF, RyR channels have increased single-channel open probability, which results in diastolic SR Ca^2+^ leak and depletion of SR Ca^2+^ content, contributing to impaired contractility and HF progression [58, 59]. We found that AVP-treated PV cardiomyocytes had significantly larger SR Ca^2+^ leak than control cells. Clinically, greater SR Ca^2+^ leak was well demonstrated in atrial myocytes in AF patients [44]. Greater Ca^2+^ leak may also contribute to a decrease in Ca^2+^_i_ transients and SR Ca^2+^ content as well. CaMKII regulates several Ca^2+^-handling proteins and has been shown to be a central regulator of excitation-contraction coupling [60], and increased CaMKII expression was found in AF [61]. CaMKII-dependent hyperphosphorylation of the RyR leads to elevated SR Ca^2+^ leak [61, 62], and triggers delayed afterdepolarization via activation of the NCX [63]. Diastolic SR Ca^2+^ leak can be amplified by NCX, triggering ectopic focal discharges or facilitating microreentry circuits promoting AF maintenance [64]. In this study, the higher pCaMKII in AVP-treated PV cardiomyocytes may result in the increased Ca^2+^ leak. The attenuation effects of KN-93 on AVP-induced Ca^2+^ leak in PV cardiomyocytes also suggests that activation of CaMKII is important for the effects of AVP on PV cardiomyocytes. In addition, the greater I_Na-Late_ in AVP-treated PV cardiomyocytes may also arise from the higher pCaMKII because activation of CaMKII is an important activator of I_Na-Late_. Enhanced I_Na-Late_ synergistically increases the risk of cardiac arrhythmias by the activation of CaMKII [17]. Accordingly, blocking the effects of AVP on PV cardiomyocytes may reduce the risk for HF-induced AF.

Our study may be limited in some respects. First, the PV cardiomyocytes received AVP for a relatively short time and AVP treatment of different duration may not have the same effects. Furthermore, AVP is usually associated with stress and pathological conditions. We only studied the effects of AVP on healthy PV cardiomyocytes, it is unclear whether our findings or theory applies to pathological settings such as HF. Finally, the details of the molecular regulation responsible for the effects of AVP in PV cardiomyocytes has not been fully elucidated.

## Conclusions

AVP increases PV arrhythmogenesis with dysregulated Ca^2+^ homeostasis through vasopressin V1 signaling.

## Data Availability

All data generated or analyzed during the current study are included in this published article.

## Abbreviations

AF: Atrial fibrillation
APs: Action potentials
AVP: Arginine vasopressin
Ca^2+^_i_: Intracellular Ca^2+^
CaMKII: Ca^2+^/calmodulin-dependent protein kinase II
DAG: Diacylglycerol
GAPDH: Glyceraldehyde-3-phosphate dehydrogenase
HF: Heart failure
I_Ca-L_: L-type calcium current
IP3: Inositol trisphosphate
NCX: Sodium/calcium exchanger
NO: Nitric oxide
PAGE: Polyacrylamide gel by electrophoresis
PVs: Pulmonary veins
Rs: Series resistance
RyR: Ryanodine receptor
SDS: Sodium dodecyl sulfate
SR: Sarcoplasmic reticulum
